# Copper-Doped Cobalt Spinel Electrocatalysts Supported on Activated Carbon for Hydrogen Evolution Reaction

**DOI:** 10.3390/ma12081302

**Published:** 2019-04-20

**Authors:** Jhony Xavier Flores-Lasluisa, Javier Quílez-Bermejo, Ana Cristina Ramírez-Pérez, Francisco Huerta, Diego Cazorla-Amorós, Emilia Morallón

**Affiliations:** 1 Departamento de Química Física e Instituto Universitario de Materiales, Universidad de Alicante. Ap. 99, E-03080 Alicante, Spain; jhonyxavier.jf@gmail.com (J.X.F.-L.); anacrami@gmail.com (A.C.R.-P.); 2 Departamento de Química Inorgánica e Instituto Universitario de Materiales, Universidad de Alicante. Ap. 99, E-03080 Alicante, Spain; javiquilezbermejo@gmail.com (J.Q.-B.); cazorla@ua.es (D.C.-A.); 3 Departamento de Ingeniería Textil y Papelera, Universitat Politècnica de Valencia. Plaza Ferrandiz y Carbonell, 1. E-03801 Alcoy, Spain; frahuear@txp.upv.es

**Keywords:** cobalt spinel, hydrogen evolution reaction, electrocatalysts, copper–cobalt oxide, microporous activated carbon

## Abstract

The development of electrocatalysts based on the doping of copper over cobalt spinel supported on a microporous activated carbon has been studied. Both copper–cobalt and cobalt spinel nanoparticles were synthesized using a silica-template method. Hybrid materials consisting of an activated carbon (AC), cobalt oxide (Co_3_O_4_), and copper-doped cobalt oxide (CuCo_2_O_4_) nanoparticles, were obtained by dry mixing technique and evaluated as electrocatalysts in alkaline media for hydrogen evolution reaction. Physical mixtures containing 5, 10, and 20 wt.% of Co_3_O_4_ or CuCo_2_O_4_ with a highly microporous activated carbon were prepared and characterized by XRD, TEM, XPS, physical adsorption of gases, and electrochemical techniques. The electrochemical tests revealed that the electrodes containing copper as the dopant cation result in a lower overpotential and higher current density for the hydrogen evolution reaction.

## 1. Introduction

The depletion of fossil fuels and the environmental problems caused by the emissions of greenhouse and other toxic gases during combustion have greatly encouraged researchers to develop technologies to extract energy from sustainable energy sources. Hydrogen is considered an ideal energy carrier to substitute current fuels due to its high specific energy, unlimited availability, the possibility of on-site production, and the evidence that hydrogen is the cleanest, non-polluting fuel [1]. Water splitting is suggested as the most appropriate way to produce hydrogen on a large scale, through the hydrogen evolution reaction (HER). Noble metal-based electrocatalysts, especially platinum-based materials, exhibit excellent activity toward the hydrogen evolution reaction (HER) but their commercial use is limited due to their low abundance and high-cost [2,3,4]. Therefore, the development of alternative non-noble metal electrocatalysts based on abundant and low-cost materials has attracted considerable attention to efficiently scale up the production of hydrogen. In order to obtain an efficient electrocatalyst towards HER, several specific properties are desirable, such as low hydrogen overpotential, no potential drift with time, good chemical and electrochemical stability, high adhesion to the support, high tolerance to poisoning by impurities, no environmental problems in the manufacturing process, and straightforward preparation at a low cost/lifetime ratio [5]. 

A wide variety of transition metal-based materials have been investigated towards HER and some of them exhibited promising results, such as dichalcogenides, metal-oxides, metal phosphides, carbides, borides, nitrides, metal alloys, and carbon-based compounds, among others [6,7,8,9]. In particular, cobalt-based compounds have emerged as interesting non-noble metal electrocatalysts because they show excellent performance for hydrogen adsorption [10]. It was shown that cobalt spinel catalysts (and those doped with another transition metal such as Ni, Fe, or Cu) exhibit good activity towards HER [11,12,13]. Specifically, the presence of Cu in the spinel structure significantly enhances the electrochemical and physicochemical properties towards HER due to the presence of Cu_2_O, which decreases the energy barrier for hydrogen adsorption [13,14,15]. Cobalt spinel materials were synthesized at a nano-size level and used for HER without any additional supporting material [15,16]. However, metal oxide nanoparticles can agglomerate, undergo deactivation of active sites, and the electrocatalyst activity can be lost. The role played by the support is, in consequence, essential for a good performance of electrocatalysts and carbon materials present several properties—such as high surface area, high conductivity or low-price—that make them ideal candidates to support these nanoparticles [2,9,17,18]. 

In the present study, we propose the development of electrocatalysts based on the doping of copper over cobalt spinel supported by activated carbon. Both copper–cobalt and cobalt spinel nanoparticles were synthesized using a silica-template method. The nanoparticles were supported by activated carbon, with a high porosity development, through physical mixture by varying the concentration of the spinel nanoparticles. The structural, morphological, and electrochemical properties were investigated with transmission electron microscopy (TEM), X-ray photoelectronic spectroscopy (XPS), X-ray diffraction (XRD), N_2_-adsorption isotherms, cyclic voltammetry (CV), and their catalytic activity towards the hydrogen evolution reaction was also analysed by cyclic voltammetry.

## 2. Experimental

### 2.1. Materials and Reagents 

Cobalt (II) nitrate hexahydrate (Co(NO_3_)ꞏ6H_2_O) (Sigma-Aldrich, ACS reagent, St. Louis, MO, USA), copper (II) nitrate trihydrate (Cu(NO_3_)ꞏ3H_2_O) (Sigma-Aldrich, 99%, St. Louis, MO, USA), potassium hydroxide (KOH) (VWR Chemicals, 85 wt.%, Prague, Czech Republic), hydrochloric acid (HCl) (VWR Chemicals, 37% vol, Fontenay-sous-Bois, France), poly(tetrafluoroethylene) (PTFE) (Sigma-Aldrich, 60 wt.%, St. Louis, MO, USA), ethanol (VWR Chemicals, 96% vol, Fontenay-sous-Bois, France), and silica xerogel (Sigma-Aldrich, TLC high-purity grade, St. Louis, MO, USA). All the reagents were used without any further purification process.

### 2.2. Synthesis Procedure

The activated carbon (AC) was prepared from a Spanish anthracite via chemical activation with KOH, following the procedure described elsewhere [19]. Briefly, the AC was synthesized by a chemical activation with KOH using an activating agent:anthracite ratio of 3:1 and by heating in a N_2_ atmosphere (400 mL/min) from room temperature to 750 °C at a heating rate of 5 °C·min^−1^. The maximum temperature was kept for 2 h. After that, the AC was washed several times with a 5 M HCl solution and with distilled water until free of chloride ions, and then dried at 110 °C for 12 h. 

Co_3_O_4_ nanoparticles were prepared by a nanocasting method with a commercial silica xerogel template [20]. First, a 1.6 M ethanolic solution of Co(NO_3_)_2_·6H_2_O was used to fill the pores of the silica sacrificial template via the incipient wetness impregnation method. The solvent was evaporated to dryness at 80 °C. The impregnation–drying cycle was repeated three times to obtain a high loading of the cobalt salt, and then the material was heat-treated in the air up to 400 °C for 4 h. After that, the cobaltite nanoparticles were obtained after dissolution of the silica template with 2 M NaOH.

CuCo_2_O_4_ were synthesized with the same procedure as described above by using Cu(NO_3_)_2_∙3H_2_O as the copper salt precursor and a Cu^2+^/Co^2+^ molar ratio of 0.5.

AC-Co_3_O_4_ and AC-CuCo_2_O_4_ materials were obtained by dry mixing the AC and the metal oxide powders in an agate mortar for 15 min. Physical mixtures containing 5, 10, and 20 wt.% of either Co_3_O_4_ or CuCo_2_O_4_ were prepared. The electrocatalysts were named considering the weight percentage of each component of the mixture as follows: AC-Co5, AC-Co10, AC-Co20, AC-CuCo5, AC-CuCo10, and AC-CuCo20.

### 2.3. Characterization Techniques

The as-synthesized oxides were characterized by X-ray diffraction (XRD) using a Cu Kα (λ = 0.1541 nm) radiation source at a step of 0.05° s^−1^ in the 2*θ* range from 30° to 80° on a Bruker D8 (Billerica, USA) Advance diffractometer. Cell parameters were calculated by a computer program using the peak position obtained after fitting the experimental range with a pseudo-Voigt function per peak plus a background line. The line-broadening analysis was performed to determine the average crystallite size [21]. 

The X-ray photoelectron spectroscopy (XPS, Sussex, UK) analysis was done with VG-Microtech Multilab 3000 equipment by employing MgKα (1253.6 eV) irradiation as the photo source. The analysis chamber pressure during scans was approximately 5 × 10^−7^ Pa and the photoelectrons were collected into a hemispherical analyzer working in the constant energy mode at a pass energy of 50 eV. The C1s binding energy from adventitious hydrocarbon was taken as a charge reference and fixed at 284.6 eV. Peak energies were determined with an accuracy of ±0.2 eV. Signal deconvolution of all XPS curves was done with mixed Gaussian–Lorentzian line shape functions after a nonlinear Shirley type background subtraction. The atomic ratio estimations were done, relating the peak areas after the background subtraction and were corrected relative to the corresponding atomic sensitivity factors. The metal oxides were characterized by transmission electron microscopy (TEM, JEOL-2010, 200 kV accelerating voltage, Akishima, Japan). The samples for TEM analysis were prepared by putting the Co_3_O_4_ and CuCo_2_O_4_ powders on a standard copper grid. The bulk composition of perovskites was analysed by energy-dispersive X-ray spectroscopy (EDX) with Bruker XFlash 3001 equipment (Billerica, USA) attached to the scanning electron microscope (SEM, Hitachi, S3000N, Chiyoda, Japan).

Surface area and porosity of chemically activated carbon and the as-prepared composites were determined by physical adsorption of N_2_ (−196 °C) and CO_2_ (0 °C), using an automatic adsorption system (Autosorb-6, Quantrachrome, Boynton Beach, FL, USA). The samples were outgassed at 250 °C under vacuum for 4 h. Nitrogen adsorption results were employed to calculate Brunauer–Emmett–Teller (BET) surface area values and Dubinin–Radushkevich (DR) micropore volumes (VDR N_2_) [22,23]. The CO_2_ adsorption data at these conditions is used to determine the narrow micropore volume (i.e., pores below 1 nm) [24]. The pore size distribution of the silica xerogel was determined from the N_2_ adsorption–desorption isotherm, applying the Barrett–Joyner–Halenda (BJH) method to the desorption branch [25].

### 2.4. Electrochemical Measurements

The electrochemical performance of the different electrodes was assessed by cyclic voltammetry (CV) at 2 mV s^−1^ in a standard three-electrode cell (Figure S1) in a 0.1 M KOH solution. The working electrodes were prepared by mixing in an agate mortar 90 wt.% as-prepared materials, 5 wt.% acetylene black, and 5 wt.% PTFE. The resulting homogenous paste-like material was cold rolled to obtain electrodes of 1 cm^2^ and around 7 mg. After that, the paste film was placed on a stainless-steel mesh, which was used as a current collector, by pressing the electrode onto the mesh under 2 ton for 5 min. A platinum wire was used as a counter electrode and a Ag/AgCl electrode as a reference electrode. However, all potentials were referred to as a reversible hydrogen electrode (RHE). The electrochemical experiments were performed with a VMP3-BioLogic potentiostat, controlled by EC-Lab software.

To calculate the gravimetric capacitance the following equation was employed:

$$C=\frac{\int_{E_{1}}^{E_{2}}i\left(E\right)dE}{\;\left(E2-E1\right)mv}\;,$$

where *E_1_* and *E_2_* are the upper (*E_2_*) and lower (*E_1_*) potential limits in which the charge is calculated from the voltammogram, *m* is the mass of the working electrode, and *v* is the scan rate. 

## 3. Results and Discussion

### 3.1. Structural and Morphological Characterization

**Figure 1:**
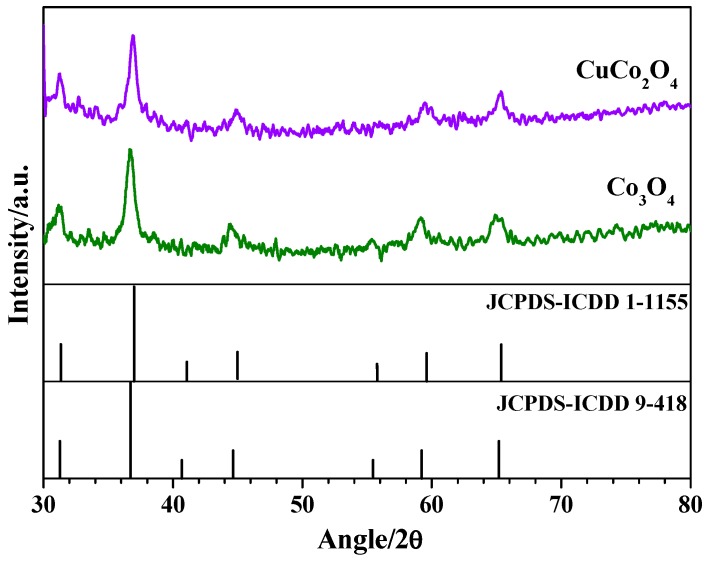
X-ray diffraction patterns of CuCo_2_O_4_ and Co_3_O_4_ nanoparticles.

The crystal structure of the nanoparticles synthesized by the silica template method was analyzed by X-ray diffraction. Figure 1 shows the X-ray powder diffraction patterns of cobaltite and copper cobaltite nanoparticles. The diffractograms of the as-prepared Co_3_O_4_ and CuCo_2_O_4_ powders exhibit reflection peaks at Bragg angles of ca. 31.25°, 36.70°, 44.45°, 55.27°, 59.21°, and 65.35°. These diffraction peaks match well with the (220), (311), (400), (442), (511), and (440) crystal planes of a cubic spinel cobaltite and a cubic spinel copper cobaltite (Fd3m [227] space group). 

Both the position and the intensities of the diffraction peaks agree with the data given in the International Centre for Diffraction Data (ICDD) cards for pure Co_3_O_4_ (JCPDS-ICDD 9-418 file) and pure CuCo_2_O_4_ (JCPDS-ICDD 1-1155 file). Moreover, the diffraction peaks for CuCo_2_O_4_ are slightly shifted to higher *2θ* values with respect to the corresponding peaks in Co_3_O_4_. The incorporation of Cu ions, small atoms with respect to the Co, into the octahedral sites of the cubic spinel Co_3_O_4_ structure contracts the lattice parameter. The lattice parameter (*a*) and the unit cell volume (*V*) of the as-synthesized cobalt oxides were calculated by Bragg equation for face-centered cubic crystals using the (220), (311), (400), (511), and (440) facets of the spinels. In addition, the coherence length of crystalline domains (i.e., crystallite size) was estimated by the Debye–Scherrer equation [20]. The unit cell parameters and the crystallite size of the oxides are summarized in Table 1.

As can be noted, the crystallite size (*d_c_*) is very similar for both materials. Furthermore, the lattice parameter and the cell volume decrease when Cu ions incorporate into the cubic spinel structure of cobalt oxide, indicating that a single phase is formed. The lattice parameter of CuCo_2_O_4_ powder is very similar to that reported in the literature by Marsan et al. [26] and Gautier et. al [27]. However, this value is smaller than those reported by other authors (values ranging from 8.11 to 8.13 were reported) [28,29,30,31]. The difference could be ascribed to the preparation method used to synthesize the spinel CuCo_2_O_4_. 

Since XRD does not detect crystal structures different from those attributed to the CuCo_2_O_4_ spinel, the SEM-EDS technique was employed to obtain a mapping of copper and cobalt elements. Figure S2 in the supporting information shows an SEM-EDX image of CuCo_2_O_4_ that confirms the lack of phase segregation. Therefore, it was concluded that pure CuCo_2_O_4_ was synthesized with this method.

**Figure 2:**
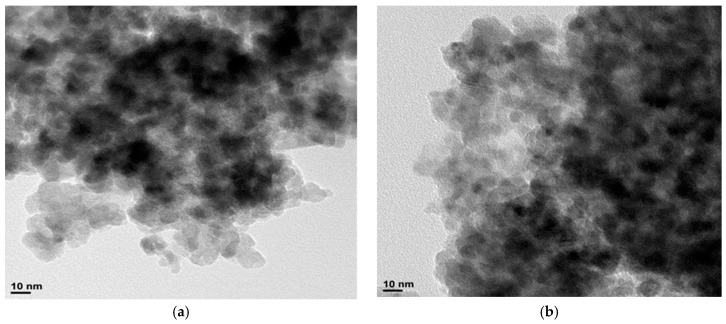
Transmission electron microscopy (TEM) images of (**a**) Co_3_O_4_ and (**b**) CuCo_2_O_4_ nanoparticles.

The morphology of the as-prepared Co_3_O_4_ and CuCo_2_O_4_ nanoparticles was evaluated by TEM (Figure 2). The micrographs show that both samples are composed of irregular nanoparticles of about 12 nm in size, in close agreement with the crystallite size determined by the Debye–Scherrer equation. The silica template exhibits mesoporosity in the 3–13 nm range (Figure S3), according to the pore-size distribution obtained by the BJH method from the nitrogen isotherm, being the maximum of the distribution at around 9 nm (Figure S3). Hence, as it was pointed out in previous works [20,32], the formation of nanoparticles takes place within the confined space provided by the mesoporous channels of the silica template.

### 3.2. Surface Analysis 

**Figure 3:**
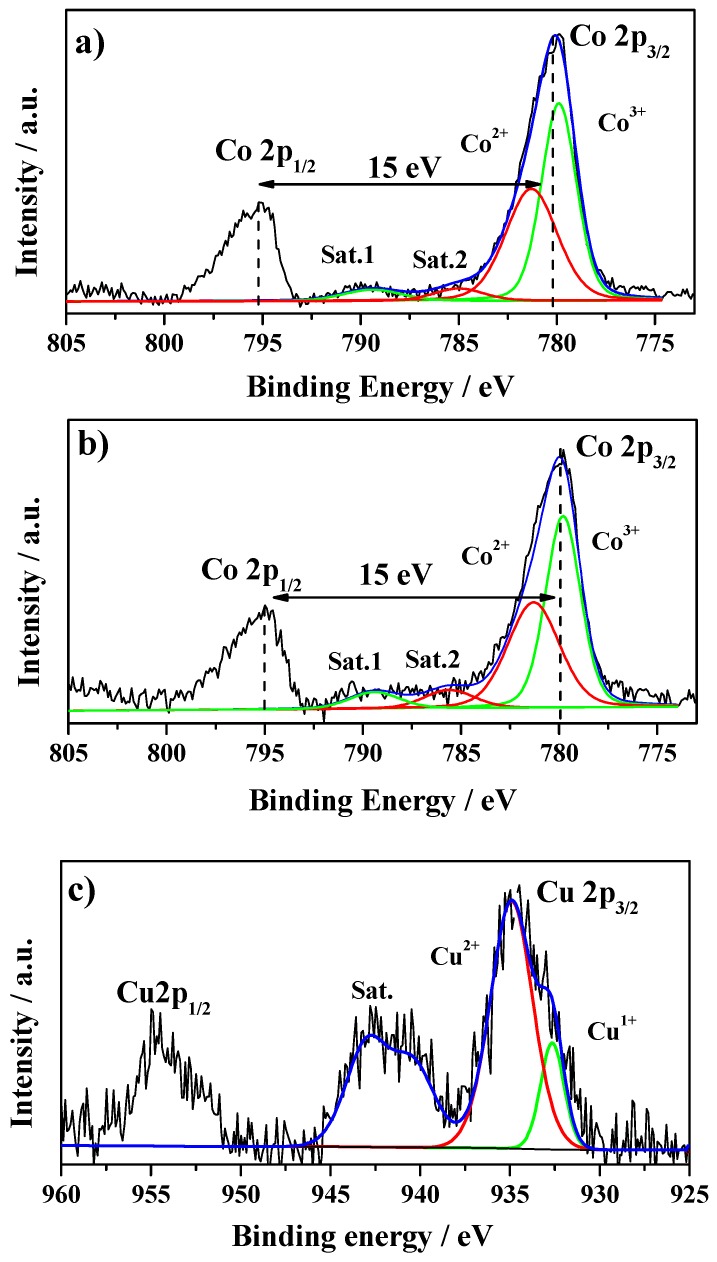
X-ray photoelectron spectra of Co 2p for (**a**) Co_3_O_4_ and (**b**) CuCo_2_O_4_, and (**c**) Cu 2p for CuCo_2_O_4_.

The surface chemical composition and the oxidation state of cobalt and copper ions in the cubic spinel powders were investigated by XPS analysis. In Figure 3, Co 2p and Cu 2p spectra recorded from Co_3_O_4_ and CuCo_2_O_4_ samples are displayed, whereas the binding energy of the photoemission lines is reported in Table 2. 

The Co 2p core-level spectrum of Co_3_O_4_ spinel (Figure 3a) shows two asymmetric main peaks separated by a spin-orbit splitting energy of 15 eV, characteristic of a mixture of Co^2+^ and Co^3+^ ions [30,33]. The Co 2p_3/2_ peak is centered at 780.2 eV and can be deconvoluted into two peaks at 779.9 and 781.3 eV. The first peak is associated with the Co^3+^ that it is contained in octahedral oxygen coordination and shows a satellite peak at 9.5 eV above the main 2p_3/2_ line. The second peak is related to Co^2+^ with tetrahedral coordination and shows a satellite peak above 3.6–6.5 eV above the main 2p_3/2_ line [34,35]. 

The Co 2p spectra of CuCo_2_O_4_ is shown in Figure 3b and reveals that the Co 2p_3/2_ and Co 2p_1/2_ peaks appear at slightly lower binding energies than those of Co_3_O_4_. The Co 2p_3/2_ signal can again be deconvoluted into two peaks centered at 779.8 and 781.3 eV that correspond to Co^3+^ and Co^2+^, respectively. According to Fierro et al. [36], the binding energy (BE) shifts to lower values in copper–cobalt oxides because of a change in the cation distribution at the spinel surface when Co^2+^ is replaced by Cu^2+^ ions into the spinel lattice.

The Cu 2p core-level spectrum of the CuCo_2_O_4_ is characterized by two main peaks ascribed to Cu 2p_3/2_ Cu 2p_1/2_ photoemission lines and a strong satellite signal between them (Figure 3c). The Cu-2p_3/2_ is characterized by an asymmetric peak centered at 934.9 eV with an intense satellite peak at 942.4 eV, and the Cu 2p_1/2_ component is characterized by a peak at ca. 954.9 eV. The peak at 934.9 eV is assigned to octahedral Cu^2+^, whereas the peak located at a BE of 932.7 eV can be attributed to tetrahedral Cu^+^, which comes from the recognized X-ray induced reduction of Cu^2+^ to Cu^+^ in the presence of adventitious carbon [26,28,30,31,36]. Therefore, the separation of approximately 20.0 eV between the main Cu 2p peaks is indicative of the existence of an open 3d^9^ shell of Cu^2+^ [37]. In addition, the I_sat_/I_main_ ratio is similar to the values reported in the literature for CuO [38] and indicates that Cu^2+^ ions on the surface of the inverse spinel adopt mainly octahedral geometry as that of CuO. The Co_3_O_4_ adopt a normal spinel crystal structure, which can be considered as a face-centred cubic (fcc) packing of oxygen anions, with Co(III) filling half the octahedral interstitial sites and Co(II) filling one-eighth of the tetrahedral sites. The substitution of Co ions by foreign divalent transition metals is known to promote an inhomogeneous distribution of cations and produce a partially inverted spinel structure, with foreign and cobalt ions occupying both octahedral and tetrahedral sites. In the CuCo_2_O_3_, the copper cation is preferentially positioned in an octahedral position, with the ratio Cu(II) tetrahedral to Cu(II) octahedral at around 0.4 [31].

Table 2 includes also the surface composition of the as-synthesized oxide materials and, as can be noted, the surface of the copper–cobalt oxide exhibits a concentration of both cations close to the expected Co:Cu ratio (2:1).

### 3.3. Textural Characterization of As-prepared Materials

As is shown in Figure S4, the activated carbon derived from the Spanish anthracite and the hybrid materials both exhibit a type I isotherm for N_2_ adsorption, which is characteristic of microporous solids. The BET surface area and the micropore volumes calculated from N_2_ adsorption data at −196 °C [V_DR_(N_2_)] and CO_2_ adsorption data at 0 °C [V_DR_(CO_2_)] are collected in Table 3. 

The BET surface area and the micropore volumes of the cobalt oxide-activated carbon and copper–cobalt oxide-activated carbon hybrid materials (AC-Co*x* and AC-CuCo*x*) were measured in order to evaluate the influence of the oxide content on the porosity of the activated carbon. It can be observed that the electrocatalysts present lower BET surface area and micropore volume compared to AC, due to the partial blockage of pores producing values of surface area lower than the corresponding amount of AC. This means that these physical mixtures do not follow the dependence predicted by the rule of mixtures [39].

### 3.4. Electrochemical Characterization and Catalytic Activity Towards HER

The electrochemical behavior of the electrocatalysts was studied by cyclic voltammetry in a three-electrode cell configuration. First, we will address the response of electrocatalysts in the potential region preceding the hydrogen evolution reaction. In this way, Figure S5 displays cyclic voltammograms recorded between 0.00 and 0.80 V for each sample, where pseudo-capacitive responses associated mainly with the formation of the electrochemical double-layer can be observed in all cases. Figure S5a reveals that an increase in the cobalt content results in a progressive loss of voltammetric charge, and therefore of specific capacitance (see Table 3 for numerical values). These values agree with the decrease in the surface area obtained. Interestingly, Figure S5b shows that when copper is present the decrease in voltammetric charge is less significant compared to the Co-containing samples.

**Figure 4:**
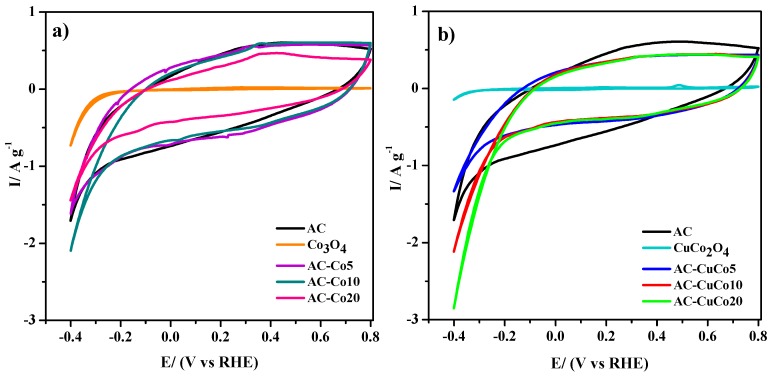
Cyclic voltammograms recorded in 0.1 M KOH showing the hydrogen evolution reaction at (**a**) undoped AC-Cox samples and (**b**) copper-doped AC-CuCox. Scan rate: 2 mV s^−1.^

The electrocatalytic activity towards the hydrogen evolution reaction of all materials was studied in the same 0.1 M KOH solution. The results are displayed in Figure 4, where cyclic voltammograms recorded between −0.40 V and 0.80 V have been depicted together. The activated carbon and the two pristine spinels (copper doped and non-doped) were included in the study for comparison purposes. It can be clearly observed that hybrid electrocatalysts obtained after mixing spinels and activated carbon improve the electrochemical performance compared to the un-supported spinels. In fact, the influence of carbon support in the catalytic activity of cobalt spinels towards the oxygen reduction reaction (ORR) was already carefully studied in chemical mixtures [41], although this has not been reported for HER. The favorable effect reported was attributed to better control over particle agglomeration induced by the supporting materials. Furthermore, Vulcan XC-72R has been used as an electric conductor to improve the catalytic properties of metal oxides [30]. According to previous studies [42], weak physical interactions exist between activated carbon and spinel nanoparticles that facilitate electron transfer between them, thus improving the overall electrical conductivity and enhancing the catalytic activity of these materials [30].

Figure 4 shows the electrocatalytic activities of all samples, as examined by cyclic voltammetry. Moreover, Table 4 shows additional data on HER electrocatalysis. Again, the control over nanoparticle sizes and the enhancement in electrical conductivity explain the higher catalytic activity towards HER shown by both doped and undoped spinels supported on activated carbon. Moreover, the substitution in the octahedral position by Cu cations could also contribute to the increase in the electrocatalytic activity.

It is known that porous carbon materials can be used to store hydrogen by the electro-reduction of water in alkaline and neutral media [43,44]. The storage occurs through reversible C–H bonds in which weakly bonded hydrogen is dominant in KOH [44]. The presence of the activated carbon can be beneficial to increase the concentration of adsorbed and dissociated hydrogen species, thus acting as a hydrogen reservoir. A tentative mechanism is presented, showing the hydrogen evolution reaction can occur in the presence of both materials. According to previous studies, HER can proceed through either Volmer–Tafel or Volmer–Heyrovsky pathways in basic mediums [45,46]:

H_2_O + e^−^ → H_ads_ + OH^−^ (Volmer)

H_ads_ + H_ads_ → H_2_ (Tafel)

H_2_O + e^−^ → H_ads_ + OH^−^ (Volmer)

H_ads_ + H_2_O + e^−^ → H_2_ + OH^−^ (Heyrovsky)

Both mechanisms involve the adsorption of H_2_O molecules and their subsequent splitting into adsorbed H atoms and OH^−^ ions. In all the materials, the high values of the Tafel slope indicate that the rate determining step is the Volmer reaction. However, this Tafel slope decreases in the AC-copper oxide electrocatalysts in comparison with the AC, indicating the electrocatalytic effect of copper in the material. In addition, the microporous activated carbon might act as a hydrogen reservoir, providing H_ads_ species to the oxide nanoparticles, where recombination into molecular hydrogen eventually occurs. This hypothesis needs further research to determine the role of the AC.

In summary, it seems that there is a relevant synergy between both the spinel nanoparticles and the carbon surface that results in an increase in the HER rate.

Besides the beneficial effect of the carbon support, Figure 4b shows that the addition of copper as a dopant leads to a noticeable increase in the catalytic activity. Obviously, the hydrogen evolution reaction takes place also on the undoped cobalt spinel, but increasing the relative amount of this active material reveals an inconsistent effect on the current at −0.4 V for hydrogen evolution (Figure 4a). On the contrary, the presence of copper improves the overall voltammetric response and increases the cathodic current at −0.4 V for this electrochemical reaction (Table 4). Such effects seem to be related to a change in the charge density at the spinel surface, which can chemisorb reactive water molecules more easily in the presence of copper ions due to the increase in oxygen vacancies and Co^3+^, thus facilitating the HER. A similar result was reported after doping with iron a cobalt spinel, for which the adsorption of water molecules was favored in Fe^3+^ in relation to Co^2+^ [12]. 

## 4. Conclusions

Hybrid materials, consisting of nanostructured cobalt or copper-doped cobalt spinels dispersed in a highly microporous activated carbon, were prepared by a dry physical mixing, characterized physicochemically, and tested as electrocatalysts for a hydrogen evolution reaction in an alkaline medium.

The use of a high surface area activated carbon as physical support improves the overall performance of cobalt-based spinel electrocatalysts. Activated carbon prevents the agglomeration of metal oxide particles and, in addition, also seems to play an active role in the HER. This is due to its well-known capacity to store hydrogen in alkaline conditions and also by an enhancement in the electrical conductivity of the oxide nanoparticles. As a consequence, a higher catalytic activity towards HER for the supported spinels compared to the un-supported materials is obtained.

It was demonstrated that the surface of copper–cobalt oxide particles shows a higher number of oxygen vacancies and Co^3+^. This surface composition results in active sites with a higher activity for hydrogen evolution than the undoped cobalt spinel, which eventually favors the formation of molecular hydrogen. Accordingly, the incorporation of copper as the dopant cation results in lower overpotential and higher current density for the target reaction. 

From the point of view of a practical application, cobalt spinel doped with copper constitutes a promising alternative catalyst for HER, since the cost of this material is significantly lower than commercial platinum-based materials.

## Figures and Tables

**Table 1:** Cell parameters and crystallite size of the as-prepared oxide nanoparticles calculated from diffraction patterns.

Sample	a (Å) ICDD Cards	a (Å)	V (Å3)	dc (nm)
Co_3_O_4_	8.09	8.10	532.72	13
CuCo_2_O_4_	8.06	8.07	526.47	14

**Table 2:** Binding energies of Co 2p and Cu 2p and surface composition obtained from X-ray photoelectronic spectroscopy (XPS) spectra of Co_3_O_4_ and CuCo_2_O_4_ nanoparticles.

Sample	Co 2p (eV)				Cu 2p (eV)				Surface Atomic Composition (at.%)		
	2p_3/2_	2p_1/2_	Sat. 1	Sat. 2	2p_3/2_	2p_1/2_	Sat.	I_sat_/I_main_	Co	Cu	Cu/Co
**Co_3_O_4_**	780.2	795.2	789.4	785	–	–	–	–	5.1	–	–
**CuCo_2_O_4_**	779.9	794.9	789.4	785.6	934.9	954.9	942.4	0.50	3.9	1.7	0.44

**Table 3:** Textural parameters obtained by N_2_ and CO_2_ isotherms for pristine AC-Co*x* and AC-CuCo*x* hybrid materials. The last column shows the gravimetric capacitance derived from cyclic voltammetry experiments.

Sample	S_BET_ (m^2^ g^−1^)	V_DR_ N_2_ (cm^3^ g^−1^)	V_DR_ CO_2_ (cm^3^ g^−1^)	*C* (F g^−1^)
AC	3310	1.30	0.76	262
Co_3_O_4_	112 [32]	–	–	1
CuCo_2_O_4_	42 [40]	–	–	2
AC-Co5	2810	1.21	0.52	261
AC-Co10	2780	1.16	0.43	247
AC-Co20	2460	1.03	0.37	154
AC-CuCo5	2910	1.20	0.51	188
AC-CuCo10	2780	1.15	0.51	178
AC-CuCo20	2220	0.92	0.40	174

**Table 4:** Electrochemical parameters of the different materials tested in the hydrogen evolution reaction.

Sample	Tafel Slope (mV/dec)	Current (A·g^−1^) at −0.4 V vs. RHE	Current (A·cm^−2^) at −0.4 vs. RHE
AC	416	−1.65	−9.5
Co_3_O_4_	132	−0.75	−5.2
CuCo_2_O_4_	124	−0.15	−1.0
AC-Co5	574	−1.60	−11.4
AC-Co10	457	−2.10	−12.8
AC-Co20	438	−1.45	−9.3
AC-CuCo5	454	−1.30	−8.1
AC-CuCo10	347	−2.15	−13.8
AC-CuCo20	300	−2.85	−14.6

## Supplementary Materials

The following are available online at https://www.mdpi.com/1996-1944/12/8/1302/s1, Figure S1: Configuration of a three-electrode cell, Figure S2: SEM-EDS elemental mapping of Co, Cu, and overlapped Co-Cu, Figure S3: BJH pore-size distribution of the silica template, Figure S4: Nitrogen adsorption isotherms at -196 °C for all the hybrid materials: (a) AC-Co*x* and (b) AC-CuCo*x* (0 ≤ *x* ≤ 20 wt.%), Figure S5: Cyclic voltammograms recorded in 0.1 M KOH for all the hybrid materials within the pseudocapacitive potential region: (a) undoped AC-Cox samples and (b) copper-doped AC-CuCox samples. Scan rate: 2 mV s^−1^.
