# Supplementary material for: Copper-Doped Cobalt Spinel Electrocatalysts Supported on Activated Carbon for Hydrogen Evolution Reaction
Source: Materials (Basel). 2019 Apr 20;12(8):1302. doi: 10.3390/ma12081302 (PMC6514974; doi:10.3390/ma12081302)
Supplement: Supplementary file 1 [file materials-12-01302-s001.pdf]

materials

Copper-Doped Cobalt Spinel Electrocatalysts Supported on Activated Carbon for Hydrogen Evolution Reaction

Jhony Xavier Flores-Lasluisa [1], Javier Quílez-Bermejo [2], Ana Cristina Ramírez-Pérez [1], Francisco Huerta [3], Diego Cazorla-Amorós [2] and Emilia Morallón [1,*]

[1] Departamento de Química Física e Instituto Universitario de Materiales, Universidad de Alicante. Ap. 99, E-03080, Alicante, Spain; anacrami@gmail.com

[2] Departamento de Química Inorgánica e Instituto Universitario de Materiales, Universidad de Alicante. Ap. 99, E-03080, Alicante, Spain; javiquilezbermejo@gmail.com (J.Q-B.); cazorla@ua.es (D.C-A.)

[3] Departamento de Ingeniería Textil y Papelera, Universitat Politècnica de Valencia. Plaza Ferrandiz y Carbonell, 1. E-03801, Alcoy, Spain; frahuear@txp.upv.es

* Correspondence: morallon@ua.es

Supporting information

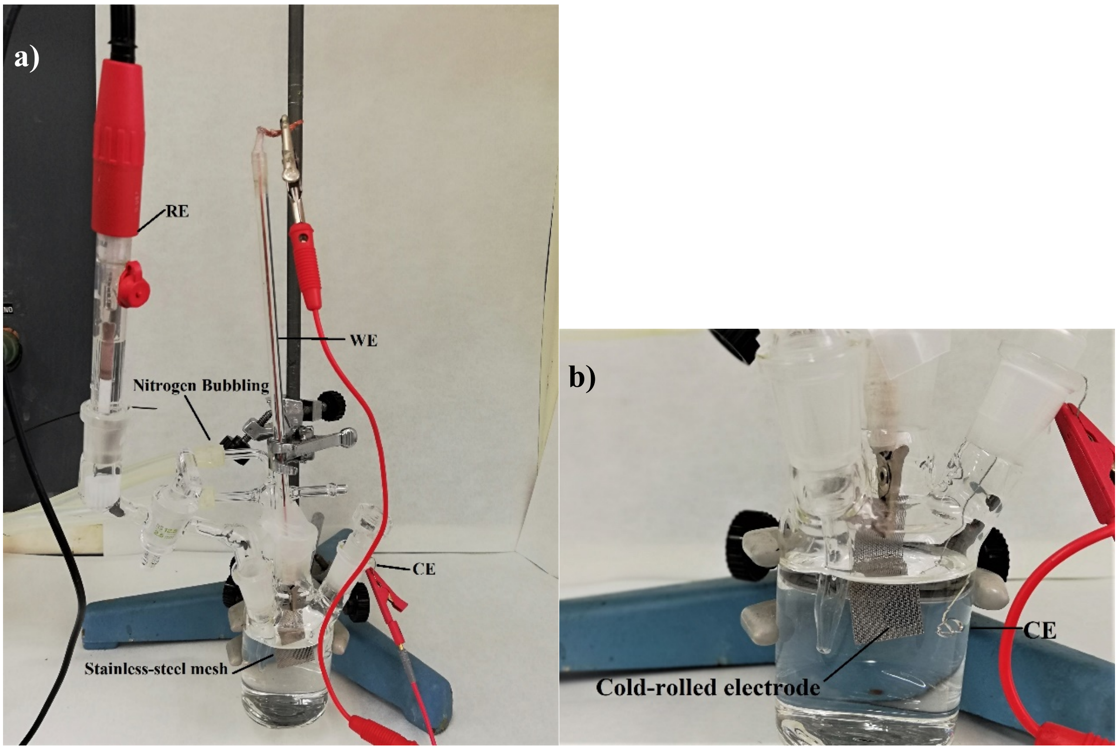

Figure S1. Configuration of a three-electrode cell.

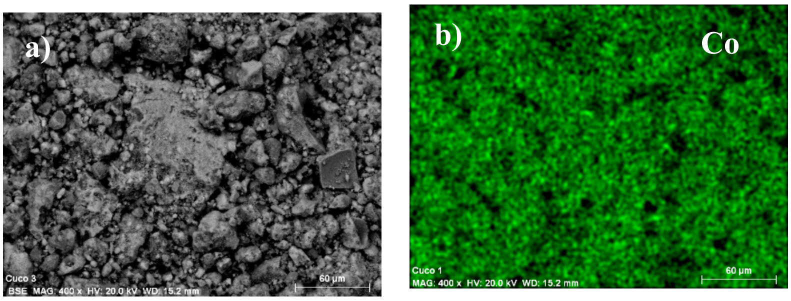

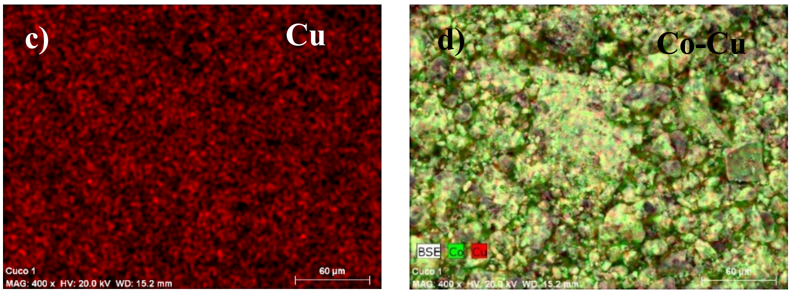

Figure S2. SEM-EDS elemental mapping of Co, Cu, and overlapped Co-Cu.

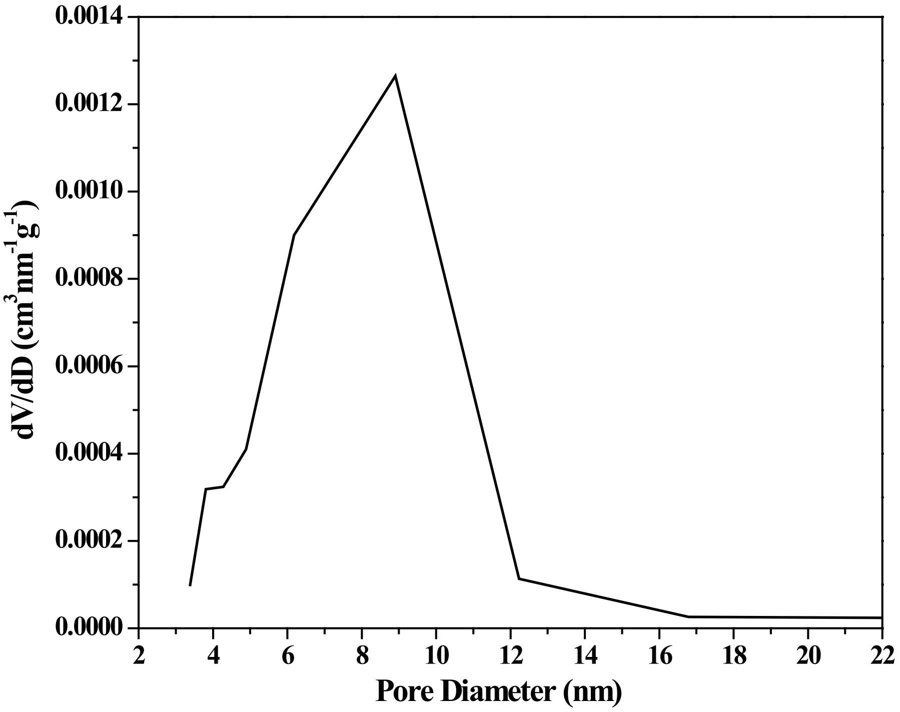

Figure S3. BJH pore-size distribution of the silica template calculated using the desorption branch of the N_2 isotherm at −196ºC.

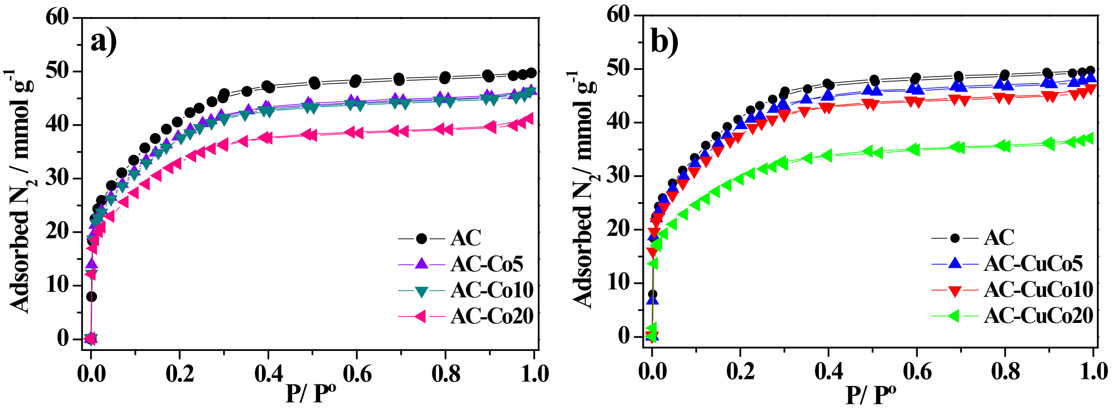

Figure S4. Nitrogen adsorption isotherms at −196 °C for all the hybrid materials: (**a**) AC-Co*x* and (**b**) AC-CuCo*x* ($0 \leq x \leq 20$ wt.%).

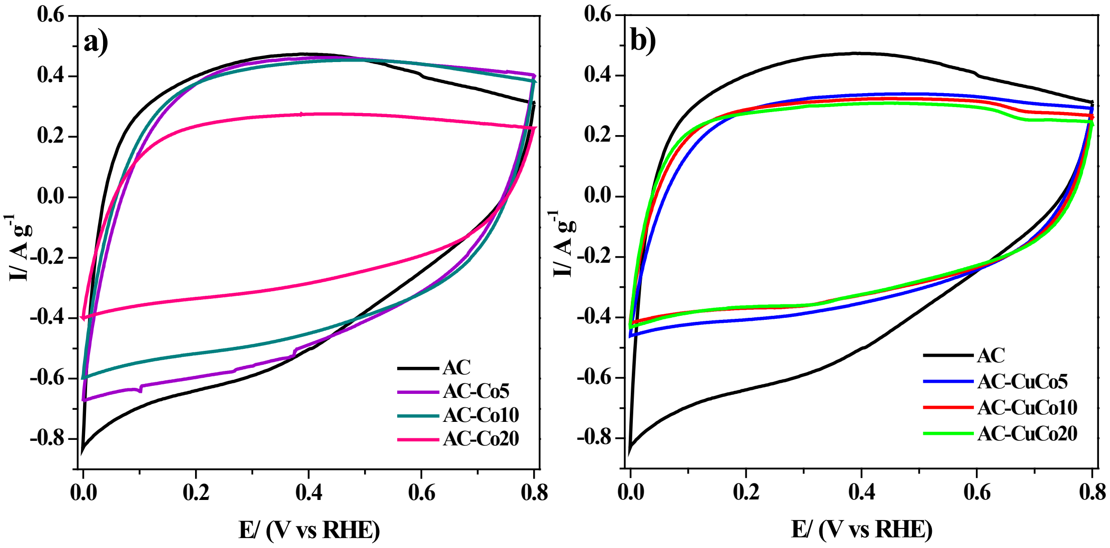

Figure S5. Cyclic voltammograms recorded in 0.1 M KOH for all the hybrid materials within the pseudocapacitive potential region: (**a**) undoped AC-Cox samples and (**b**) copper-doped AC-CuCox samples. Scan rate: 2 mV $s^{-1.}$
